# Recruitment of Adolescent Young Carers to a Psychosocial Support Intervention Study in Six European Countries: Lessons Learned from the ME-WE Project

**DOI:** 10.3390/ijerph20065074

**Published:** 2023-03-14

**Authors:** Francesco Barbabella, Lennart Magnusson, Licia Boccaletti, Giulia Casu, Valentina Hlebec, Irena Bolko, Feylyn Lewis, Renske Hoefman, Rosita Brolin, Sara Santini, Marco Socci, Barbara D’Amen, Yvonne de Jong, Tamara Bouwman, Nynke de Jong, Agnes Leu, Daniel Phelps, Elena Guggiari, Alexandra Wirth, Vicky Morgan, Saul Becker, Elizabeth Hanson

**Affiliations:** 1 Department of Health and Caring Sciences, Linnaeus University, 39182 Kalmar, Sweden; 2 The Swedish Family Care Competence Centre (NKA), Strömgatan 13, 39232 Kalmar, Sweden; 3 Anziani e Non Solo Società Cooperativa Sociale, Via Lenin 55, 41012 Carpi, Italy; 4 Department of Psychology, University of Bologna, Viale Berti Pichat 5, 40127 Bologna, Italy; 5 Faculty of Social Sciences, University of Ljubljana, Kardeljeva pl. 5, 1000 Ljubljana, Slovenia; 6 School of Nursing, Vanderbilt University, Godchaux Hall 179, 461 21st Ave S, Nashville, TN 37240, USA; 7 School of Education and Social Work, University of Sussex, Falmer, Brighton BN1 9RG, UK; 8 The Netherlands Institute for Social Research (SCP), Postbus 16164, 2500 BD The Hague, The Netherlands; 9 Centre for Socio-Economic Research on Aging, IRCCS INRCA-National Institute of Health and Science on Aging, Via Santa Margherita 5, 60124 Ancona, Italy; 10 Vilans—The National Centre of Expertise for Long-Term Care in The Netherlands, Churchilllaan 11, 3527 GV Utrecht, The Netherlands; 11 Institute for Biomedical Ethics, Medical Faculty, University of Basel, Bernoullistrasse 28, 4056 Basel, Switzerland; 12 Department of Health, Kalaidos University of Applied Sciences, Gloriastrasse 18a, 8006 Zurich, Switzerland; 13 Faculty of Health and Well-being, University of Winchester, Winchester SO22 4NR, UK; 14 Careum, Pestalozzistrasse 3, 8032 Zurich, Switzerland; 15 Carers Trust, 32–36 Loman Street, London SE1 OEH, UK; 16 Faculty of Health and Education, Manchester Metropolitan University, Manchester M15 6BX, UK

**Keywords:** young carers, adolescent young carers, recruitment, co-design, COVID-19, psychosocial support, cluster-randomised controlled trial, intervention study

## Abstract

Young carers provide a substantial amount of care to family members and support to friends, yet their situation has not been actively addressed in research and policy in many European countries or indeed globally. Awareness of their situation by professionals and among children and young carers themselves remains low overall. Thus, young carers remain a largely hidden group within society. This study reports and analyses the recruitment process in a multi-centre intervention study offering psychosocial support to adolescent young carers (AYCs) aged 15–17 years. A cluster-randomised controlled trial was designed, with recruitment taking place in Italy, the Netherlands, Slovenia, Sweden, Switzerland and the United Kingdom exploiting various channels, including partnerships with schools, health and social services and carers organisations. In total, 478 AYCs were recruited and, after screening failures, withdrawals and initial dropouts, 217 were enrolled and started the intervention. Challenges encountered in reaching, recruiting and retaining AYCs included low levels of awareness among AYCs, a low willingness to participate in study activities, uncertainty about the prevalence of AYCs, a limited school capacity to support the recruitment; COVID-19 spreading in 2020–2021 and related restrictions. Based on this experience, recommendations are put forward for how to better engage AYCs in research.

## 1. Introduction

Young carers are children and young people (<18 years old) who provide regular and substantial care to ill or disabled family members [1]. Recent estimations about the prevalence of young carers in Europe indicate that about 7–8% of all children carry out substantial amounts of caring [2,3,4,5,6]. Despite the progressively growing interest of researchers, practitioners and policy makers in this target group [7,8], evidence and awareness by stakeholders (e.g., schools, health and social services) remain limited in most countries, with the exception of the United Kingdom (UK) where the phenomenon started being investigated and addressed in the early 1990s [7]. Otherwise, in most countries, policies, legislative frameworks and welfare systems do not directly recognise the role and needs of young carers, relying on “non-specific” legislation concerning education, health and social care, safeguarding and child protection, and family [8,9,10,11,12]. In fact, the formal recognition of young carers and availability of dedicated services, as in the UK, exist in only a few countries [13,14,15,16,17,18,19,20]. By formal recognition, we mean, for instance, the identification of young carers by policy makers and institutions as a target group with specific needs and rights to obtain support by the community.

Previous research has brought attention to the difficulty of unequivocally stating whether a young person is providing regular and/or substantial care (thus to be considered as a young carer) or not. In this respect, it is more appropriate to consider care activities provided by young individuals in the light of a continuum of care ranging from caring about the person to caring for the person, as opposed to a binary condition (i.e., being a young carer or not) [21,22].

Low awareness and preparedness by formal and educational services, as well as the challenges related to defining young carers, lead to a range of problems when researchers and practitioners aim to identify young carers for research or providing support [23,24]. Young carers often risk being relatively invisible, hidden or underserved by educational and social services, thus receiving little attention and being difficult to reach through formal channels [25,26].

In general terms, reaching young carers and involving them in research is complex [24]. Challenges are particularly exacerbated with children and minors who need a parent or guardian’s approval to participate in research, since they may have limited familiarity and understanding about research and its purposes and effects [27], as well as about the implications and impact on caring [28]. Young carers’ perception of possible social stigma and fear of being bullied by peers if participating in support initiatives may also play a role and negatively influence attitudes regarding participation [28,29].

Furthermore, selective participation mechanisms exist for recruitment, due to the following: structural factors, e.g., a lower likelihood that a sub-group of the target population with certain demographic or socio-economic characteristics are considered, found or concretely involved by researchers in a study, and agency-related factors, e.g., a lower likelihood of a certain sub-group to be interested and willing to participate in a research study. These mechanisms usually produce biases based on the fact that people from lower socio-economic status, minority groups, rural areas or with poorer health are less likely to be invited and ultimately be involved in research [30]. Despite evidence existing on selective participation focused on adult and older individuals [31], little is known about this effect in younger age groups; yet, it can be assumed that this mechanism somehow also occurs for children and young people.

Phelps [32] suggested that, in addition to the barriers to participation that children and young people may experience generally, there are additional barriers that young carers are likely to face as a consequence of their caring responsibilities, including increased time constraints and a lack of transport. Phelps [32] also highlighted recruitment challenges as a consequence of young carers not accessing dedicated support services nor identifying as a carer themselves.

Among young carers, a specific sub-group of adolescent young carers (AYCs) aged 15–17 years are an under-investigated target group. This age group of adolescents deserves more attention in research, since they find themselves in a delicate, transitionary phase between childhood and adulthood [33,34,35,36]. At this age, several developmental processes are happening at the same time (e.g., the development of personal and social identities), together with progressive end-of-school obligations and possible access to higher education or the labour market. Furthermore, young people in this age group are usually covered by child legislation in Europe, despite their proximity to becoming adults. Previous research in the field has barely focused on such a target group [36]. This is a subject in need of further exploration, particularly because of the gap in formal support provision for adolescent aged young carers who “age out” of child services [33,34,35,36].

The aim of this article is to report and analyse the recruitment process in an intervention study designed to provide psychosocial support to adolescent young carers (AYCs) aged 15–17 years in six European countries. The intervention study was conducted within the broader “Psychosocial support for promoting the mental health and well-being among adolescent young carers (AYCs) in Europe” project (ME-WE), funded by the European Union under the Horizon 2020 programme (grant agreement no. 754702) [36]. Ultimately, the goal is to offer lessons learned and recommendations from our experiences of conducting research among this target group, supporting researchers and practitioners working with AYCs. The ME-WE project filled gaps in the literature on the phenomenon of AYCs, representing, to the best of our knowledge, the most systematic and comparative study concerning AYCs worldwide.

## 2. Materials and Methods

The ME-WE project included a wide range of quantitative and qualitative research, co-design and knowledge translation activities dealing with the phenomenon of AYCs in Europe. Below, we describe, review and critique the methods and recruitment-related aspects of the trial evaluating the newly designed psychosocial intervention for AYCs. An overview of the ME-WE project, activities carried out and results is available elsewhere [8,9,10,11,12,33,34,36,37,38,39,40].

### 2.1. Design

The ME-WE psychosocial intervention was designed as a cluster-randomised controlled trial (C-RCT) design, with a two (arm) by three (times) repeated measures factorial design [34]. Cluster randomisation was chosen over individual randomisation to minimise the risk of contamination [41]. The study was conducted in six European countries, namely Italy (IT), the Netherlands (NL), Slovenia (SI), Sweden (SE), Switzerland (CH) and the UK. 

Clusters consisted of AYCs attending the same school (in Slovenia, Sweden and Switzerland) or living in the same geographical area (e.g., neighbourhood) (in Italy, the Netherlands and the UK). Clusters were randomised to the ME-WE intervention or wait-list control arm using an online number generator. To achieve a certain degree of blinding, AYCs were informed that the study aimed to investigate the effects of different support strategies on AYCs’ well-being and were not offered detailed information about the respective other trial arm during the study.

Outcomes were measured at the individual level. Primary outcomes were psychological flexibility; mindfulness skills; resilience; subjective mental and physical health; quality of life; impact of caring; and social support. Secondary outcomes were self-reported school, training or work experience performance and attendance. Control variables included caring activities, overall amount of caring and likes and dislikes of caring. The outcomes for the ME-WE intervention arm were compared with the wait-list control arm from the baseline (pre-intervention) through post-intervention and 3-month follow-up (3MFU). After the 3MFU, participants in the wait-list control arm were offered the same programme as the intervention arm.

The ME-WE C-RCT was registered as a trial in 2019 (ClinicalTrials.gov Identifier: NCT04114864).

More details on the intervention design are described elsewhere [34,36].

### 2.2. Intervention

The ME-WE primary prevention intervention was developed by refining the existing DNA-V protocol [42] to the specific needs and experiences of AYCs aged 15–17. DNA-V is an evidence-based model based on a combination of Acceptance and Commitment Therapy (ACT) and positive psychology and is underpinned by contextual behavioural science. The model aims to help young people cope with challenges, stress and change. In the framework of ME-WE, the model was adapted to be used with AYCs and it was co-designed with AYCs and professionals (e.g., psychologists, teachers, youth workers, health professionals) in the framework of the Blended Learning Networks (BLNs) carried out during the project. BLNs are heterogeneous communities of practice that enable the voices of users and multi-stakeholders to be heard and that lead to shared learning [43]. In the ME-WE project, each country set up a BLN, which included between 8 to 14 participants (AYCs, comprising former young carers, and professionals) with the goal of contributing to the project implementation by providing their expert knowledge and experiences. 

Participants of clusters allocated to the ME-WE intervention arm attended seven weekly 2 h group sessions, with a follow-up meeting after 3 months from the end of the intervention. Groups were comprised of 2 to 9 AYCs (with the exception of Switzerland and Slovenia where individual interventions were held with one participant; in addition, in Slovenia there was one group with 23 participants). Group dynamics represent an important part of the intervention. To facilitate a proxy of group dynamics, the facilitators were instructed to also step into the participant role in cases where groups were small.

All sessions had a similar structure (objectives, ice-breaker, central activity/ies and final activity). After a first introductory session regarding the DNA-V model, sessions 2, 3 and 4 introduced the main concepts used. Session 5 dealt with values and values-oriented action and session 6 focused on attaining psychological flexibility and self-compassion (i.e., the ability to approach personal suffering and failures with openness and awareness, showing self-kindness [44]). The closing session 7 focused on building strong social networks. At the end of sessions 2, 4, 5 and 6, participants were provided with exercises to perform at home, between one meeting and the next one, to practice the skills acquired during the session in everyday life. In the follow-up session, participants reinforced the skills learned and discussed their experiences with the intervention.

Two different methods were originally followed in the delivery of the ME-WE intervention (that is, prior to the COVID-19 pandemic): a fully face-to-face approach (adopted by Italy, Slovenia and UK), and a blended approach that combined face-to-face and online sessions delivered via video-conferencing tools and a dedicated ME-WE mobile app (adopted by Sweden, Switzerland and the Netherlands) which was co-designed with AYCs at earlier stages of the ME-WE European project.

It was originally planned that AYCs in the control group would perform ice-breaker and team-building activities during three meetings organised to correspond with the three assessment points with the aim of collecting outcome measures data.

The interventions were carried out in the six countries during the period June 2019–March 2021.

### 2.3. Ethics Approval and Informed Consent

The study protocol and related documentation were assessed and approved/expert opinions were provided (in accordance with national legislation) by competent ethics committees in each of the six countries during 2019. All participants were engaged in the study on a voluntary basis in accordance with the Declaration of Helsinki [45].

All participants were provided with a plain-language statement describing the general purpose of the study. They were informed about the voluntary nature of their participation and their right to withdraw from the study at any time without having to provide a reason and without any adverse consequences. 

Information letters and informed consent forms were handed over to AYCs, one set for themselves and, when required by legislation, one set for their parents or legal guardians, to be returned later on. Written self-consent was obtained from participant AYCs and, when required by legislation, also from their parents or legal guardians. In Sweden and Switzerland, parental consent is not a legal requirement for young people aged 15–17 years. For 14-year-olds (see Section 2.5 and Section 2.6 about the reasons for their involvement), parental consent was sought and obtained from parents or guardians. In Slovenia, parental consent is required for AYCs aged 15. Where parental consent was not required according to national legislation, then participants’ legal representatives were provided with information about the study and the young person’s participation, wherever deemed feasible or appropriate. 

Data were processed in compliance with both national laws on data protection and the EU General Data Protection Regulation (GDPR) 2016/679 to guarantee the respondents’ confidentiality and privacy.

The amendments introduced due to the COVID-19 pandemic received formal ethical approvals and/or detailed opinions (as appropriate according to national legislation) from the previously consulted ethics review boards in all six countries. They were informed that, due to the COVID-19 situation, it was necessary to adapt the methodological approach by replacing all personal meetings (group sessions) of the intervention with online meetings (see Section 2.8 below) and therefore to deliver the entire intervention online. The registered study protocol was updated with protocol amendments at clinicaltrials.gov following ethics approvals.

For more details, see the Institutional Review Board Statement at the end of the article.

Ethics issues, especially those concerning the implementation of the intervention during the pandemic, were also carefully monitored by the project’s external International Advisory and Ethics Board (IAEB) until the project end.

### 2.4. Sample

During the study protocol preparation, we conducted an average sample size estimation for each country (considering the cluster-level randomisation) [46], based on the assumed prevalence rate of AYCs in the target population of 15–17 years.

The minimum total sample sizes obtained from this procedure were as follows (Table 1): 80 AYCs in Italy and Sweden, 76 AYCs in Slovenia, 102 AYCs in Switzerland, 112 AYCs in the Netherlands and 142 AYCs in the UK. These numbers were revised after the onset of the pandemic and adaptations of the study protocol.

### 2.5. Inclusion and Exclusion Criteria

To be eligible for the study, the following inclusion criteria were established: (1) aged between 15 and 17 years at the recruitment stage; (2) caring for family member(s) (e.g., parents, siblings, grandparents) or significant other(s) (e.g., friends, schoolmates or neighbours) with a disability, chronic physical and/or mental health condition or substance use issue and/or problems related to old age [1,20]. Exclusion criteria were as follows: (1) concurrently participating in psychotherapies or mindfulness-based interventions/programs; (2) having started a new psychotropic medication within the past 30 days or planning on starting or changing psychotropic medication during the course of the study; (3) limited knowledge of the local language. The inclusion and exclusion criteria were assessed at the screening interview through a phone call or a face-to-face meeting conducted by the research team members.

In response to the COVID-19 pandemic and to boost the recruitment of AYCs, the Swedish Ethical Review Authority and the Vrije University Amsterdam Research Ethics Review Committee approved the target group to be expanded from 15–17 years to 14–17 years. In the UK, the competent research ethics committee approved the target group to be expanded to 14–18-year-olds. 

### 2.6. Compassionate Cases

All participating countries allowed participants who did not meet the inclusion criteria to attend the ME-WE groups. They were identified as AYCs during recruitment and were interested in participating in the intervention. They expressed a need for support, but no alternatives for support other than the ME-WE-groups were available for them at that particular time or place. During the COVID-19 pandemic, the issue of compassionate use became more relevant as almost all regular support for young people was or remained cancelled due to the pandemic restrictions.

In most of the cases they had not met the inclusion criteria as a consequence of their age, i.e., being 14 or 18 years old. In the UK and Sweden, those on psychotropic medication and those either in current receipt of a psychotherapeutic intervention or mental health counselling or planning to receive such therapies during the course of the ME-WE C-RCT study were also deemed compassionate cases.

Access to the ME-WE groups was allowed on a case-by-case assessment. National Clinical Trial Managers (CTMs) requested permission for inclusion from the project’s CTM and Ethics, Gender and Data Manager (EGDM). Informed consent procedures were applied as standard in this C-RCT study for AYCs aged 15–17. Furthermore, for participants below the age of 15, informed consent of a parent or guardian was obtained. Compassionate cases might have completed the evaluation questionnaires at any of the three assessment points, but their data were excluded from the formal evaluation of the intervention.

### 2.7. Recruitment

The recruitment of AYCs was carried out between April 2019 and September 2020. All countries employed cluster-targeted recruitment methods aimed to reach a convenience sample. The efforts were invested to overcome the known difficulties to reach and involve AYCs, especially in countries where there is no formal recognition of their role. Thus, convenience approaches were designed in each country, based on the specific context (e.g., availability and willingness to support the study by schools, health and social services and non-profit organisations), by the leading research partner organisation. 

The recruitment of AYCs was performed in either schools only (in Slovenia, Sweden and Switzerland) or geographical areas (in Italy, the Netherlands and UK) by also partnering with, in addition to schools, community-based service organisations (e.g., community health and social service agencies and organisations, youth welfare agencies, carer-related or disease-specific non-governmental organisations (NGOs) and young carers charities). In Sweden, a large school campaign was conducted. In Slovenia and Switzerland, recruitment was expanded from initially targeting schools to dormitories and campuses, respectively, as well as to a wider range of additional stakeholders (i.e., from the health and the social sectors, including NGOs). In Italy, the majority of participants were identified and recruited with the mediation of a public or private health or social care service and through condition-specific associations and NGOs working with young persons. The UK differed from the other ME-WE partner countries because they have a widespread network of young carers support services already in place. Thus, recruitment efforts in the UK focused firstly on recruiting appropriate young carers projects to the ME-WE study and secondly on recruiting AYCs to take part in the study. A variety of recruitment methods were adopted by the research teams in each country. Among these, the collaboration with local stakeholders (e.g., school staff, professionals from health and social sectors) was fundamental in order to promote the participation in the study among young people [34]. In the Netherlands, research assistants—often former young carers—were trained by the ME-WE project members and assisted schools with the recruitment, screening, implementation and evaluation of the RCT study on site. 

A screening interview (either in person or via telephone) against eligibility criteria was conducted by the research team in every country. During the screening, the AYCs were given the opportunity to ask questions regarding the project. In the UK, AYCs and their parents/legal guardians took part in a screening call with the ME-WE group member and the UK Clinical Trial Manager. 

Sweden and Switzerland endeavoured to turn to the randomisation of individual participants, since their recruitment method did not pose a risk for spill-over effects. As outlined above, due to COVID-related interruptions and a slowing down of the recruitment of trial participants, Sweden and Switzerland launched national social media recruitment campaigns. In Sweden, the recruitment was supplemented with advertising via short films on social media and further information through the project’s website. In Switzerland, recruitment was also expanded with the development of a social media strategy, through blogs, websites, newsletters and social media, including the creation of a new Instagram account and using paid promotions. 

Following the COVID-19 pandemic outbreak, recruitment and enrolment were performed remotely in the six countries. Screening interviews to assess the eligibility of participants were carried out exclusively by telephone or video-conferencing applications (e.g., Zoom, Microsoft Teams). Written parental (or guardian) consent and self-consent to be involved in the study were collected by email or recorded (by video-conferencing applications).

### 2.8. Adaptation of Study Protocols

The COVID-19 pandemic posed (from March 2020 onwards) considerable challenges to the trial study in all six countries. Largely due to ethical considerations put forward by the project’s International Advisory and Ethics Board (IAEB), namely the possibility to continue to offer support to AYCs during the pandemic via their participation in the ME-WE project, combined with more pragmatic concerns to avoid costly trial closures, deviations from the original study protocol were considered unavoidable by the research team. Amendments to the study protocols were prepared and submitted for approval to competent local ethics committees. To comply with the restrictions and precautionary measures introduced at national levels, the study was virtualised, to include remote enrolment, screening, consent and data collection, as well as a fully online delivery of the ME-WE intervention [47,48].

Partners amended the original study protocol in the second half of 2020, which involved a few changes to the intervention delivery, while the core contents of the intervention remained the same. Specifically, the fully face-to-face method, planned for Italy, Slovenia and the UK was replaced by online sessions using secure video-conferencing instruments, allowing for visual presentations of participants and session materials (e.g., Zoom, Microsoft Teams). In addition, the prior “blended delivery approach” in use in Sweden, Switzerland and the Netherlands and including a combination of face-to-face and online sessions supported by an app developed ad hoc for the project and co-designed with AYCs was replaced solely by online meetings using the ME-WE mobile app and supported with the Zoom video-conferencing system.

### 2.9. CONSORT Flowchart

**Figure 1:**
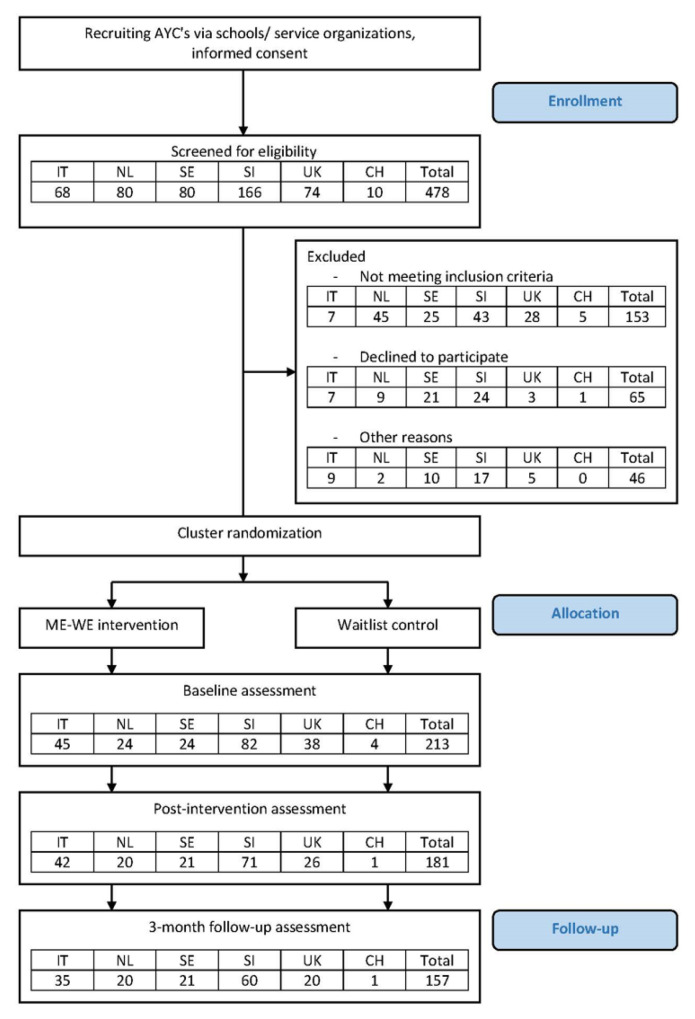
CONSORT flow diagram for the original study protocol.

The Consolidated Standard of Reporting Trials (CONSORT) 2010 statement extended to cluster trials was followed in this study [49]. The research flow diagram is presented in Figure 1. 

## 3. Results

### 3.1. Recruitment Process in the Six Countries

There were considerable difficulties in recruiting AYCs to the trial. Below, we present a summary of recruitment efforts and outcomes for each country.

#### 3.1.1. Italy

Initially, in Italy, researchers attempted to recruit AYCs in high schools and municipalities but without success, due to the low level of visibility of AYCs. Thus, it was decided to try to engage AYCs with the mediation of public or private health and/or social care services and through condition-specific associations and NGOs working with young persons. In this case, it was a trusted professional that took the initial contact with the parents/guardians to ask for permission and then invited the AYC to participate. Following this, a member of the research staff further contacted the parents and the AYC to provide all the necessary details, respond to questions and organise the collection of the consent forms before proceeding to the screening. The majority of participants were identified in this way. 

A minority of participants contacted the research team after having found leaflets or online information about the project. In this case, after an initial contact with the AYC, a phone contact with the parents/guardians was sought to provide all the information, secure consent and eventually organise the logistics of the group. The recruitment process took place mostly before the COVID-19 pandemic, while some participants were recruited later but following the same process (i.e., with the mediation of trusted professionals).

#### 3.1.2. The Netherlands

In the Netherlands, the recruitment was carried out through schools and care support centres. 

The first wave of recruitment involved the following: (a) A total of nine schools (three high schools and six schools in vocational education) entered the study, of which six schools in vocational education cancelled participation before the start of the intervention (such cancellations were due to the timing of the intervention in the schools’ calendar, so that it was too close to the examination period, which would have meant students being too stressed with studying for exams and not having time for participating in the ME-WE intervention); (b) A total of 19 informal care support centres were approached. Of these, four support centres accepted to take part in the study by contributing to recruitment efforts, but two of them dropped out before the start of the intervention. In March 2020, immediately prior to the COVID-19 outbreak in this country, a second wave of recruitment was initiated: 17 care support centres were engaged, of which 13 centres dropped out before the start of the intervention. In June 2020, recruitment was conducted online by the care support centres using social media posts, at (online) activities during the Dutch National Week of the Young Carer (1–7 June 2020) and through inviting young carers on their contact lists.

#### 3.1.3. Slovenia

In Slovenia, the recruitment strategy was built on liaising with high schools as well as with non-governmental organisations and care services dedicated to the disability and mental health fields concerning young people and adults. The majority of the recruitment was carried out via school presentations and direct recruitment within classrooms during the first wave and in the second wave the same approach was applied in student dormitories (these are institutional facilities for students that prefer to stay at the place of school during week days, for instance if they live in remote areas and would otherwise have to commute to school). The COVID-19 pandemic led to the need for recruitment in the third and fourth waves to move to online platforms used by schools to communicate with students. During the second and fourth wave, schools also directly recruited participants via school advisors. COVID-19 hindered the recruitment of participants especially for the intervention groups.

#### 3.1.4. Sweden

In Sweden, a school campaign was conducted, which reached out to 1081 students and 354 school staff in twelve schools. In the first intervention wave, Sweden adopted a blended delivery approach. In the second wave, with COVID-19 restrictions underway, all recruitment was conducted via advertisements and videos on social media, reaching out to over 100,000 young people and professionals. With these considerable efforts and resources, the research team was able to recruit a few potential participants per week, but if the young people had to wait for joining a group they dropped out. The priority was therefore to create and start groups as soon as possible, and a decision was made to wait with the control group to make sure that there would be at least some groups in Sweden. During the pandemic, Sweden adopted a fully online approach with the use of the ME-WE mobile app and supported by the Zoom video-conferencing system.

#### 3.1.5. Switzerland

In Switzerland, recruitment was initially entirely and directly through schools. Recruitment was expanded in December 2019, and whilst it continued through the schools (with additional direct recruitment on school campuses), recruitment also took place through a wider range of additional stakeholders from the health and the social sectors, including NGOs. Recruitment was also expanded with the development of a social media strategy. In total, 24 schools participated, thanks to a robust effort of approaching 590 stakeholders and 27 schools (through presentations, emails, phone calls, fliers and posters in schools, as well as contacts and meetings with school directors, staff and educational departments), which covered an estimated 16,000 young people. During the COVID-19 pandemic, recruitment activities on school campuses became restricted and then cancelled altogether. The focus of the recruitment therefore shifted further to phone calls and email communications via the schools and additional stakeholders and linked to social media recruitment. 

#### 3.1.6. United Kingdom

In the UK, there were two waves of recruitment. In the first wave, Carers Trust (a UK-based charity) led the recruitment of the young carers projects to the study through its network of young carers support services. Carers Trust sent out informational leaflets to its network of hundreds of young carers projects across England. Three network partners were chosen because of their interest and perceived ability to achieve the target sample size. They were tasked with finding schools that would permit the ME-WE sessions to take place. Before COVID-19 struck, 13 schools were recruited for participation. However, once the pandemic started, the UK research team moved to an online mode of delivery. In Spring 2020, the team decided to expand its recruitment effort and performed an outreach to its wider network partners in England to find other organisations willing to take part in the project. A final total of eight young carers projects were recruited to participate in the study.

### 3.2. Recruitment Results

There were considerable differences in recruitment outcomes across countries. A large number of screen failures (young people who, among those screened, did not match with the screening criteria) are noted in the Netherlands and Slovenia, and these countries, together with Sweden, also recorded a prominent number of withdrawals (Table 2). Initial attrition rates (withdrawals over the young people positively screened) were as follows: 11.5% in Italy; 25.7% in the Netherlands; 19.5% in Slovenia; 38.2% in Sweden; 20.0% in Switzerland; 6.5% in the UK; and 20.0% in total.

“Final sample sizes” in Table 3 correspond to the number of participants included in the quantitative analysis at the baseline, after excluding compassionate cases (i.e., young carers who did not meet the eligibility criteria, such as those who were younger than 15 years or older than 18 years) and dropouts (i.e., those who initially started the intervention but then dropped the participation). Details on the implementation of intervention and control groups per recruitment wave per country can be found in Table 4 and Table 5.

Numbers of completed evaluation assessments at each assessment point per study arm per country considered in the quantitative analysis are shown in Table 6. These figures represent participants who met inclusion criteria as outlined in the study protocol. It should be noted that participants who attended less than 70% of the intervention group sessions were excluded prior to the statistical analyses (included in the “dropout” section in Table 3). Furthermore, the final sample sizes presented here exclude both compassionate cases in some countries, as well as 14-year-olds (involved in Sweden, the Netherlands and the UK) and 18-year-olds (in the UK).

### 3.3. Target Sample Size vs. Final Sample Size: Challenges

Due to difficulties in the recruitment process, recruitment to the control groups was often delayed and thus they contain a smaller number of AYCs than the intervention group/s. Additionally, the amount of reported data from the control groups differs between the countries, making a comparison more difficult. 

As can be seen from Table 7, it proved unfeasible to reach the original target sample sizes in any of the partner countries, except Slovenia. In two countries (Italy and Sweden), the total final sample size was moderately lower, 41.4 and 56.3% of the target size, respectively. In the other two countries (The Netherlands and the UK), the final sample size was even lower (21.4 and 26.8% of the target size, respectively), whereas in Switzerland it was impossible to use data for any quantitative analysis due to the very low number of AYCs recruited (only four in total, 6.9% of the total target). For Switzerland and Sweden, the trial could not be conducted as a C-RCT in a strict sense, due to low recruitment rates and the related adaptation of the methodology decided during the study.

The methodological limitations of the study include the recruitment difficulties, screen failures and high levels of dropouts, both prior to the group start (e.g., in Sweden and Switzerland), as well as during the intervention (e.g., in Slovenia and the UK). While acknowledging all the challenges and efforts put in place, it should be recognised as a limitation that the number of participants to the study was lower than originally expected and that this influenced the power of the quantitative analysis results. The total final sample size (107 in the intervention group and 106 in the control group) was less than half of the original target sample size (263 in the intervention group and 263 in the control group) required to perform in-depth statistical analyses. Although the amended study protocols accommodated smaller sample sizes, the methodological limitation remains.

The recruitment process proved to be challenging in all partner countries, for a variety of reasons common to many of the countries involved, in addition to the COVID-19 pandemic:

- Low levels of awareness and invisible young carers (in Italy, the Netherlands, Sweden and Slovenia) imply significant efforts in engaging relevant stakeholders, as it requires a prior time investment in raising awareness on the issue of young carers and on the need to support them before being able to engage stakeholders in the recruitment process.
- Uncertainty about the prevalence of AYCs (in Italy, the Netherlands and Sweden): The prevalence of young people recognised as young carers in partner countries (between 6 and 8%) was an estimation, which brought uncertainty about where and how to approach AYCs in the society. The recruitment channels used made it necessary to reach a very high number of young people in order to have the opportunity to recruit the target number of AYCs.
- Willingness of AYCs to participate in the study (in Italy, the Netherlands, Slovenia and Switzerland): Many partners underlined that it is challenging to convince participants in the age range of this study to participate in this research, both because of a lack of recognition of themselves as carers and because of their reluctance to participate in activities where they have to expose themselves and their emotions. In Switzerland, for example, some AYCs who dropped out from the intervention reported that they preferred to use their (limited) free time for other types of activities, e.g., sport.
- Limited capacity of schools (including school staff) to participate in a research project (in the Netherlands, Sweden, Switzerland and the UK): The involvement of schools, while strategic in most of the countries involved, proved to be challenging. On the one hand, the decision-making processes in schools can be complicated and often require approvals from different levels of the organisation. On the other hand, schools and teachers are often involved in a variety of projects and activities, which does not allow them much time to dedicate to other actions, such as externally funded research, such as the ME-WE project. The COVID-19 pandemic posed further challenges to school staff, who needed to prioritise other things ahead of the ME-WE project.

### 3.4. Mitigation Measures Implemented

Project partners implemented several measures along the recruitment process, aimed at mitigating the abovementioned challenges and the risk of involving too few participants:

- Extra efforts in communication activities: All partners implemented additional communication activities to reach-out to a larger number of potentially interested young carers, through online channels (e.g., social networks) as well as being physically present in schools or in other places where young people commonly gather.
- Involvement of more stakeholders/partner organisations, as well as former young carers and young people: All partners identified and involved other organisations (e.g., non-profit organisations) and stakeholders (e.g., former young carers) that could help them to reach out to AYCs, engage them through the organisation of meetings and involve young people as research assistants in recruitment.
- Logistical measures: Many partners worked to provide as much logistic support as possible to their participants. This meant both scheduling sessions at times and places that could make it easier for young carers to attend and providing transportation to meeting venues.
- Organisational measures: Some partners executed contingency measures to help catch up with initial delays, such as running two sessions per week instead of one.
- Methodological adaptation: A mixed-method process evaluation was ultimately adopted with the engagement of stakeholders in all six partner countries. This allowed the project research team to complement quantitative information on the impact of the intervention together with a deeper understanding of the challenges encountered during the recruitment and implementation of the ME-WE intervention in each country.

## 4. Discussion

The recruitment of participants for the ME-WE trial study has certainly proved that AYCs are a hard-to-reach target group for researchers and practitioners. A lower number of participants were recruited and fully engaged, despite the significant recruitment efforts that were made and additional efforts spent. 

In order to approach AYCs and ask them to participate in the study, the first challenge was to identify them in the general population. This task was carried out in partnership with local non-profit organisations, as well as institutional services such as schools and social care. In addition, recruitment campaigns (e.g., through social media) were implemented in some cases, targeting children and young people. Since in most countries the phenomenon of AYCs Is not formally recognised and both practitioners have low awareness about this target group and AYCs themselves have low self-awareness of their role, there was a need to train field workers about the issue and to understand with them the best channels to exploit for recruitment. The identification of AYCs was difficult due to all these factors and resulted in a limited number of AYCs being approached (n = 478, 90.9% of the total target sample size) and involved in the study after screening, compassionate cases and withdrawals (n = 260, 49.4% of the total target sample size).

The second challenge was to keep AYCs interested while waiting for their ME-WE group to start and subsequently to keep them engaged in the study until the end. Despite a high retention rate (82% of those starting the study), a further reduction (18%) in AYC cases available for the quantitative analysis occurred, leading to a total final number of 217 cases (41.3% of the total target sample size). However, in some countries there was a higher number of dropouts (especially in Slovenia and the UK), which suggests that specific problems were faced during the intervention implementation. The need to fill in online surveys with a relatively large battery of sensitive questions might have deterred some AYCs from participating in the evaluation. Furthermore, the long waiting period of 3 months after the end of the intervention may have contributed to reducing the AYCs motivation to complete the questionnaire at T2.

Overall, the ME-WE trial study was conducted in specific and highly challenging times under COVID-19 restrictions of physical and social distancing and periodic lockdowns in most participating countries. The COVID-19 restrictions led to a temporary complete halt of the recruitment process in all six countries, thus leaving partners to endorse social media campaigns (Switzerland, Sweden), which were based on an individual recruitment strategy. The delivery of the intervention itself was severely hindered by the outbreak of the COVID-19 pandemic, as the Netherlands and Slovenia were in the field during the first lockdown period that affected Europe. The intervention was moved fully online; however, there was a long pause in Slovenia, for example, in waiting for all intervention materials to be adjusted to an online delivery, which resulted in increased dropout rates. Due to the transition to online platforms during lockdown, some participants were unable to continue participating owing to technical barriers (see the ethical considerations below). The recruitment barriers prevented partners from reaching sufficient sample sizes and, consequently, it was not feasible to perform a complete quantitative analysis both at the national level as well as the cross-national level.

The restricted age group (15–17 years) that was targeted could have been another limiting factor to identifying AYCs. Minors in this group are in a transition phase of their personal development from childhood to adulthood and may be subject to a variety of social influences (e.g., perceived social stigma of attending a psychosocial support intervention), as well as practical constraints (e.g., limited time to attend the intervention sessions due to school, family and social and care commitments). Moreover, as AYCs are more likely to come from socially disadvantaged groups, family circumstances and other issues may have prevented some from joining in and finding sufficient time and resources to dedicate to such a project.

Moreover, in general, the study design of interventions such as the one proposed by the ME-WE project deserves careful consideration. RCTs are widely used in medical and health-related research, especially for drug discovery and efficacy evaluation, but their use in social research with psychosocial interventions such as the ME-WE intervention are far less common. The reasons include ethical considerations but also practicalities of the process and how to conduct rigorous (reliable) RCTs in social/community settings with a “hard to reach” and invisible group such as AYCs, where professionals and other gatekeepers have little awareness of the “target group” [25]. However, we argue that there is a need for RCTs to test and determine whether a psychosocial intervention “works” or has some positive/beneficial outcomes. If one cannot test efficacy, then it is not feasible to show that interventions have an effect or not, or worse still that they could make things worse. Indeed, we acknowledge that researchers do need RCTs alongside a battery of other research and evaluation tools in social research. The ME-WE RCT highlights the complexities of recruitment and of conducting such an exercise, especially when COVID-19 struck globally and forced significant changes in the protocol and in AYC responses, withdrawals and retainment. This study reveals the trials and tribulations of this work, highlighting the need for flexibility and agility in the conduct of the RCT whilst retaining the need for rigour and in dealing with compassionate cases (those AYCs who otherwise would have had no support at all). In this way, our study has enabled reflection and learning to support others who may want to replicate or conduct other RCTs in this field.

Despite recruitment challenges and study limitations (some explained also in Hanson and colleagues [36]), the ME-WE intervention study stands out as the first-ever comparative RCT with AYCs. Recent research has been mostly focusing on observational investigations of specific aspects of caring in young age—for instance, young carers’ experiences [23], resilience [50], socio-economic consequences [51] and psychosocial positive and negative effects [52]—with limited samples in a single site or country. A large cross-sectional study with 673 young carers was conducted in 21 European countries (based on the 2014 wave of the European Social Survey) [53], still with observational purposes. To the best of our knowledge, the ME-WE dataset is currently among the most rigorous ones at the global level among interventional studies. The unique inclusion of six nations, with different awareness and response levels regarding AYCs [7,8,9], is ground-breaking on a pan-European level, and it is the first time that the DNA-V programme has had dedicated use with an AYC population. The goals of the ME-WE intervention model—to strengthen AYCs resilience, contribute positively to their mental health and well-being, and to mitigate the negative impact of psychosocial and environmental factors—have been achieved to some extent in this study [36].

As a result of the impact of the COVID-19 pandemic on the study, the ME-WE intervention model, which was originally designed for a face-to-face approach and a blended face-to-face and app approach, was shown to be adaptable and well functioning in a fully online approach [36]. One advantage of online groups is that they are independent of geographical distances and thus can include AYCs who live far from each other and at a distance from the group facilitators. Thus, the ME-WE model can be offered to all AYCs regardless of where in the country they live [36].

The mixed-methods evaluation ultimately implemented relies on a robust and sufficient amount of quantitative and qualitative data. These latter ones suggest some evidence of promising results that can shape future interventions and further research in this field. Although, due to several methodological limitations, the data should be interpreted with caution, the results suggest some positive impacts. Despite all the challenges faced in the course of the project, the ME-WE model provided a benefit to the mental health and overall well-being of the AYCs, as expressed by the AYCs through their own first-hand accounts. 

## 5. Conclusions

Drawing from the lessons learned from the recruitment process in our study and inputs received by stakeholders during the project (the IAEB and the Eurocarers Young Carers Working Group), we developed a set of recommendations and practical suggestions for researchers and practitioners dealing with AYCs, which may help them to better approach, involve and retain AYCs in future research studies:

- Time schedule and setting: Have the intervention at school and during school hours but also consider having it in a different setting that is not identifiable as dedicated to a psychosocial intervention. The venue should avoid being too formal, as this might scare off participants. It should rather be a place that can take the pressure away. Adapt the time schedule and setting to the needs and preferences of the participants’ situation. Provide food and snacks.
- Young-people-friendly and appropriate remuneration: Make agreements with local service providers/NGOs (as appropriate according to the local context where the research is taking place) and encourage them to offer pro bono fun activities (e.g., bowling or cinema tickets, as appropriate in the specific country context). Alternatively, build in a small budget line within proposed action grants/research proposals focusing on the topic of young carers to be able to appropriately remunerate participant YCs for their time and efforts in a study and as a way of respecting and thanking them for their involvement in the research.
- Logistics: Provide AYCs with as much practical help as possible to attend (e.g., pick them up and offer rides home, using accessible venues and offering technical devices for online meetings (such as headphones if needed)).
- Organisation: In light of the COVID-19 experience and of the difficulties encountered in keeping AYCs engaged, use a blended approach—in person and online—for planning the AYCs intervention. This blended approach could also promote the participation of AYCs with a high burden of care activities and/or school commitments.
- Communication: Use online tools; have a YouTube influencer or another famous person (ideally with young caring experience) sponsoring the intervention; or, when going to schools to raise awareness on young carers and to present the project, focus more on the relationship aspect. When presenting in a country with low levels of public awareness on young carers, specific attention should be paid to clearly communicating about the target group for intervention, while trying to keep a positive and empowering attitude towards AYCs. On a practical note, provide AYCs with reminders of upcoming sessions and check with AYCs that they are able to attend, and, if not, how they could be supported to attend.
- Create trust and build positive relationships: The key is to take the fear away and to let young carers trust the intervention team. It is suggested to envisage a session n. 0, that is a preliminary meeting before the actual start of the intervention. This session would simply be an opportunity for young carers to meet the intervention team, build relationships and break the fear and they could invite their friends to this session. This most likely could consist of a fun activity, e.g., a gathering with music and food. A session 0 would also help to build trust and good relationships with AYCs from the beginning.
- Flexible participation: It is important to enable AYCs to participate to the best of their situation. For example, if they attend online from settings with restricted privacy (other family members present), taking related precautions such as switching off their camera and writing their responses in the chat as appropriate. In the event of absence from a session, there is a need to follow up with the missing AYC/s and facilitate their continuation in the intervention by offering a missed session/s at another suitable date and time. In order to maintain the interest of recruited AYCs, keep in touch with them regularly during the waiting period between the screening interview and the start of the intervention. This also applies to the period between the end of the intervention and the evaluation follow-ups, taking adequate precautions for avoiding affecting the investigated long-term effects of the intervention.
- Network of key stakeholder groups: It would be valuable to use a 6-12 months period in the design to build a network of key stakeholder groups, such as professionals, representatives from civil societies and young people themselves that have direct contact with youth/young carers. For instance, in some countries a network of carers organisations could already be in place, but in others contacts with schools and/or care support centres might need to be established and several meetings at different levels (managements, teachers, mentors, etc.) are necessary before schools take a decision to participate or not.
- Design: Consider using a design with different waves, so that one school could hold two different intervention groups (in two consecutive school years). This could increase the willingness of schools to participate, because there is a pay off in the second year (efficiency of delivery, knowledge on the procedures, etc).
- Improving recruitment: It is useful to offer training and promotional materials for schools, carer organisations and health and social services. By having them “on board” the chances to recruit participants are likely to be higher.
- Co-designed intervention: Since a psychoeducational intervention may make young carers feel “different” or “wrong” in comparison with their peers, it is suggested to further tailor the ice-breaking activities around the young participants’ interests and capabilities. For example, if young participants are keen on the arts, they could agree to produce a short theatre piece or song about their caring experience.

## Figures and Tables

**Table 1:** Minimum composition of the national sample size (initial estimation).

Country	AYCs in the Intervention Group (n)	AYCS in the Wait-List Group (n)	Total (N)
Italy	40	40	80
Sweden	40	40	80
Slovenia	38	38	76
Switzerland	51	51	102
Netherlands	56	56	112
United Kingdom	71	71	142
Total	296	296	592

**Table 2:** Recruitment of participants to the ME-WE trial for each partner country.

	Total Recruitment *	Screen Failures **	Withdrawals ***	Actual Total Enrolment ****
Italy	68	7	7	54
The Netherlands	80	45	9	26
Slovenia	166	43	24	99
Sweden	80	25	21	34
Switzerland	10	5	1	4
UK	74	28	3	43
Total	478	153	65	260

* Total number of young persons who applied to the study and were screened. This number includes participants with informed consent (with an exception of a few participants in Switzerland and the Netherlands—they are included in this number but excluded from the analysis). ** Total number of young people who, among those screened, did not match with the screening criteria (this number also includes compassionate cases). *** Total number of AYCs eligible for participation (applied and were screened positively) but who eventually did not start the intervention. **** Total number of participants who are considered enrolled in the intervention (i.e., meeting inclusion criteria and attending at least one session/evaluation point (this number does not include compassionate cases, but it does include dropouts).

**Table 3:** Number of AYCs participating in the intervention, number of compassionate cases, number of dropouts and final sample size per study arm and per country.

		Included in the Intervention	*Of Whom: Compassionate Cases*	*Of Whom: Dropouts*	*Of Whom: Final Sample Size*
Italy	Intervention	38	2	9	27
	Control	19	1	0	18
The Netherlands	Intervention	19	1	2	16
	Control	15	7	0	8
Slovenia	Intervention	59	9	17	33
	Control	49	0	0	49
Sweden	Intervention	28	2	10	16
	Control	8	0	0	8
Switzerland	Intervention	6	3	3	3
	Control	1	0	0	1
UK	Intervention	37	18	4	15
	Control	33	9	1	23
Total	Intervention	187	35	45	110
	Control	125	17	1	107

**Table 4:** Description of the recruitment waves by country for the intervention group: time and type of delivery, number of groups delivered and total number of participants per wave and per country.

Country	Wave	When	Delivery	# Groups/Individual Sessions	# Participants
Italy	1	October 2019	Face-to-face	4	20
	2	July 2020	Online	2	12
	3	October 2020	Online	1	6
				Total	38
The Netherlands	1	January 2020	F2F and APP *	3	10
	2	June 2020	Online and APP	3	9
				Total	19
Slovenia	1	October 2020	Face-to-face	3	8
	2	February 2020	F2F and online	5	36
	4	September 2020	Online F2F and online	4	15
				Total	59
Sweden	1	March 2020	F2F and APP + online and APP	1	4
	2	September 2020	Online and APP	6	24
				Total	28
Switzerland	1	May 2020	Online and APP	1	3
	2	September 2020	Online and APP	1	3
				Total	6
United Kingdom	1	June/July 2020	Online	9	31
	2	September/October 2020	Online	2	7
				Total	38

* Last session conducted online.

**Table 5:** Description of the recruitment waves by country for the control: time of delivery with total number of participants per wave and per country.

Country	Wave	When	# Participants
Italy	1	October 2019	13
	2	July 2020	2
	3	October 2020	4
		Total	19
The Netherlands	1	January 2020	9
	2	June 2020	6
		Total	15
Slovenia	1	October 2020	0
	2	February 2020	0
	3	June 2020	37
	4	September 2020	12
		Total	49
Sweden	3	November 2020	8
		Total	8
Switzerland	2	September 2020	1
		Total	1
UK	1	July 2020	30
	2	September/October 2020	6
		Total	36

**Table 6:** Number of completed evaluation assessments at baseline (T0), post-intervention (T1) and follow-up (T2) for each country as included in the quantitative analysis.

		T0	T1	T2
Italy	Intervention	27	27	23
	Control	18	15	12
The Netherlands	Intervention	16	15	15
	Control	8	5	5
Slovenia	Intervention	33	28	26
	Control	49	43	34
Sweden	Intervention	16	14	14
	Control	8	7	7
Switzerland	Intervention	3	0	0
	Control	1	1	1
UK	Intervention	15	12	8
	Control	23	14	12
Total	Intervention	110	96	86
	Control	107	85	71

**Table 7:** Final target sample size and final sample size.

	Final Target Sample Size			Final Sample Size			Total Target vs. Total Final (%)		
	Intervention	Control	Total	Intervention	Control	Total	Intervention	Control	Total
Italy	40	40	80	27	18	45	67.5%	45.0%	56.3%
The Netherlands	56	56	112	16	8	24	28.6%	14.3%	21.4%
Slovenia	38	38	76	33	49	82	86.82%	128.9%	107.9%
Sweden	29	29	58	16	8	24	55.2%	27.6%	41.4%
Switzerland	29	29	58	1	3	4	3.4%	10.3%	6.9%
UK	71	71	142	15	23	38	21.1%	32.4%	26.8%
Total	263	263	526	107	106	213	40.7%	40.3%	40.5%

## Data Availability

The ME-WE project reports, deliverables and outputs are publicly accessible in the project website: https://me-we.eu (accessed on 25 October 2022). Open research data have been published in connection to related published papers.
